# Supplementary material for: Complex Structure of Lasiopodomys mandarinus vinogradovi Sex Chromosomes, Sex Determination, and Intraspecific Autosomal Polymorphism
Source: Genes (Basel). 2020 Mar 30;11(4):374. doi: 10.3390/genes11040374 (PMC7230192; doi:10.3390/genes11040374)
Supplement: Supplementary file 1 [file genes-11-00374-s001.zip › Table S1.doc]

**Table S1.** Comparison between KII and KIII females by the reproductive success over a three-month period for the two most common female karyomorphs

| Measure of reproductive success | karyomorph III | karyomorph II | Statistics and p-value |
| --- | --- | --- | --- |
| Proportion of the females that gave birth (%) | 12 of 15  (80) | 12 of 13  (92) | Fisher’s exact test:  p=0.600 |
| Number of litters, X±SE | 1.75±0.22 | 1.91±0.25 | t=0.48; p=0.635 |
| Number of born offspring, X±SE | 5.91±0.87 | 6.50±0.99 | t=0.45; p=0.656 |
| Number of weaned offspring, X±SE | 4.25±0.96 | 4.54±0.06 | t=0.21; p=0.824 |
