# Supplementary material for: Complex Structure of Lasiopodomys mandarinus vinogradovi Sex Chromosomes, Sex Determination, and Intraspecific Autosomal Polymorphism
Source: Genes (Basel). 2020 Mar 30;11(4):374. doi: 10.3390/genes11040374 (PMC7230192; doi:10.3390/genes11040374)
Supplement: Supplementary file 1 [file genes-11-00374-s001.zip › Table S2.doc]

**Table S2**. Chromosome-specific microdissected probes of *L. m. vinogradovi* and syntenic regions in mouse genome assembly GRCm38

| Probe | Specimen | Microdissected chromosome | Homologous mouse chromosome | Coordinates of the beginning | Coordinates of the end |
| --- | --- | --- | --- | --- | --- |
| L2 | LMAN19f | neo-X_1_ | chr18 | 20818375 | 90530293 |
|  |  |  | chrX | 4060107 | 169958428 |
| L3 | LMAN19f | neo-X_2_ | chr13 | 71139264 | 119756963 |
|  |  |  | chr15 | 3262310 | 32405356 |
|  |  |  | chr15 | 93642559 | 103558715 |
|  |  |  | chr18 | 20818375 | 90530293 |
|  |  |  | chrX | 4060107 | 169958428 |
| L5 | LMAN15m | neo-Y | chr18 | 20818375 | 90530293 |
| L8 | LMAN15m | neo-X1 | chr18 | 20818375 | 90530293 |
|  |  |  | chrX | 4060107 | 169958428 |
| L11 | LMAN14f | neo-Y | chr18 | 31562946 | 90530293 |
| L13 | LMAN14f | neo-Y dist | chr18 | 20818375 | 90530293 |
| L31 | LMAN5f | neo-X_2_ | chr13 | 71139264 | 119756963 |
|  |  |  | chr15 | 3262310 | 32405356 |
|  |  |  | chr15 | 93642559 | 103911984 |
|  |  |  | chr18 | 20818375 | 90530293 |
|  |  |  | chrX | 4060107 | 169958428 |
| L33 | LMAN14f | neo-X_2_ | chr13 | 71139264 | 119756963 |
|  |  |  | chr15 | 3262310 | 32405356 |
|  |  |  | chr15 | 93642559 | 103558715 |
|  |  |  | chr18 | 20818375 | 90530293 |
|  |  |  | chrX | 4060107 | 169958428 |
